# Daily Peer Interactions and Mood in Black and Latiné Youth: The Roles of Friends and Parents

**DOI:** 10.3390/bs16050683

**Published:** 2026-04-30

**Authors:** Sunhye Bai, Dawn P. Witherspoon, Miglena Y. Ivanova, Tiyobista M. Maereg, Carlos F. Almeida, Emely Covarrubias, Dulce M. Gonzalez, Griselda Martinez, Mayra Y. Bámaca

**Affiliations:** 1 The Ballmer Institute for Children’s Behavioral Health, Department of Psychology, University of Oregon, Portland, OR 97211, USA; 2 Department of Psychology, The Pennsylvania State University, University Park, PA 16802, USA; dpw14@psu.edu (D.P.W.);; 3 Psychological Sciences, University of California, Merced, CA 95343, USAmbamaca@ucmerced.edu (M.Y.B.); 4 School of Social Policy & Practice, University of Pennsylvania, Philadelphia, PA 19104, USA; 5 Center for the Study of Health and Risk Behaviors, Department of Psychiatry and Behavioral Sciences, University of Washington, Seattle, WA 98195, USA

**Keywords:** adolescents, peers, race–ethnicity, racial–ethnic homophily, parent–youth interactions, mood, emotions, daily diaries, PVEST

## Abstract

To advance our understanding of the social and emotional development of minoritized youth in new destination areas, we examined whether same-day links between positive interactions with friends and mood vary by two contextual factors—(a) the racial–ethnic composition of adolescents’ close friends, and (b) the quality of parent–adolescent interactions—among Black and Latiné adolescents living in United States. Data were obtained from two daily diary studies, one of 36 Black adolescents (44% male; 11 to 17 years old; *M* = 13.65, *SD* = 2.29), and one of 21 Latiné adolescents (52% male; 11 to 14 years old; *M* = 12.76, *SD* = 1.00). Across 10 school days, youth completed afternoon reports of positive interactions with friends and bedtime reports of mood and positive parent–youth interactions; the race–ethnicity of the friend group were assessed at baseline. Multilevel models separated day-level from person-level effects and tested moderation by the racial–ethnic composition of friends and parent–youth interactions. For Black adolescents with few same-race friends (0 to 1 out of max 3), when youth reported more positive friend interactions, they reported lower positive mood. The opposite was true for Black youth with more same-race friends; when youth reported more positive friend interactions, they reported more positive mood at the end of the day. Among Black youth with higher mean levels of positive parent interactions, when youth reported more positive friend interactions, they reported lower negative mood. Results from the analysis of Latiné youth did not support study hypotheses. Findings extend cultural–ecological–transactional frameworks of development by showing that the short-term emotional yield of supportive interactions with friends hinges on contextual vulnerabilities and assets, such as peer group composition and family relationship quality, especially for Black youth.

## 1. Introduction

Adolescence is marked by the expansion of social contexts beyond the family, with friends and peers playing increasingly central roles (La Greca & Harrison, 2005). Friends and peers are important for promoting social and cognitive development, as well as psychological adjustment (Sebanc et al., 2016; Way & Pahl, 2001). Meta-analytic evidence shows that friendship experiences, both positive and negative, are reliably associated with adolescents’ depressive symptoms and loneliness, underscoring the emotional salience of peer relationships across development (Schwartz-Mette et al., 2020). Recent intensive longitudinal research further suggests that fluctuations in friendship dynamics co-occur with changes in adolescents’ internalizing symptoms (Schacter et al., 2023). Together, this work underscores the importance of examining peer experiences as dynamic contributors to adolescents’ emotional lives.

However, much of the extant literature on adolescent friends and peers is largely based on White American samples, with comparatively less attention given to the relational processes shaping emotional well-being among racially or ethnically minoritized youth (Padilla et al., 2024), who develop and engage with peers within racialized contexts that have mental health consequences (Way & Chen, 2000; Benner et al., 2018). Furthermore, the relatively limited research with minoritized youth is often conducted in traditional destination areas for immigrants or diverse metropolitan cities (e.g., New York City, Miami, Phoenix, Los Angeles) (Azmitia et al., 2009; Brown et al., 2018; Padilla et al., 2024; Price, 2012). Minoritized youth living in new destination areas—non-traditional immigrant settlements with few co-ethnics (Marrow, 2020; Massey & Capoferro, 2008)—may be exposed to unique neighborhood and school characteristics that shape their relationships with their peers and parents. For example, neighborhood and school racial–ethnic compositions (Witherspoon et al., 2023) may be more diverse, creating greater marginalization and weakened social bonds for parents while simultaneously providing more opportunities for intergroup contact that can facilitate cross-race and cross-ethnic friendships, especially for youth (Graham, 2018; Chan & Benner, 2025). This potential gap between parents’ and youth’s experiences may shape caregiver–youth relationship quality, positively (e.g., strengthening bonds due to shared experiences) or negatively (e.g., creating conflict due to differing perceptions of outgroup members). Given these realities, prior findings about the roles of peers, friends, and parents may not generalize to minoritized youth in new destination areas (Brown et al., 2018; Price, 2012).

Moreover, past research on daily peer experiences often focuses on negative interactions with friends and peers (Bai et al., 2017; Chen et al., 2024), and a better understanding of the short-term implications of positive peer interactions for minoritized youth in new destination areas is needed to inform strength-based and inclusive approaches to health promotion. Thus, the objective of this study was to examine the benefits of supportive, understanding, and complementary experiences with friends—hereafter referred to as positive friend interactions—on the daily mood of Black and Latiné adolescents in a new destination area. We investigated how same-day associations between positive friend interactions and mood vary across two contextual factors: the racial–ethnic composition of an adolescent’s friend group, and the quality of parent–adolescent interactions.

### 1.1. Theoretical Frameworks

The current study is rooted in the Phenomenological Variant of Ecological Systems Theory (PVEST) (Spencer et al., 1997; Spencer, 2008), which emphasizes context sensitivity and how people make meaning of their experiences. The theory is inclusive, time-centered, and process-oriented, highlighting the interactions between risks and protective factors (i.e., net vulnerability) and their impacts on youth coping responses. These interactions contribute to youth’s emergent identities and, consequently, their long-term outcomes. In this study of Black and Latiné youth in a new destination area, it is critically important to understand their normative experiences, whether stressful or not, and ascertain how these youth make meaning of their peer and family interactions, which create varying levels of net vulnerability. We seek to empirically explore this dynamic and transactional process allowing for non-deterministic thinking about racial–ethnic minoritized youth’s experiences.

**Figure 1:**
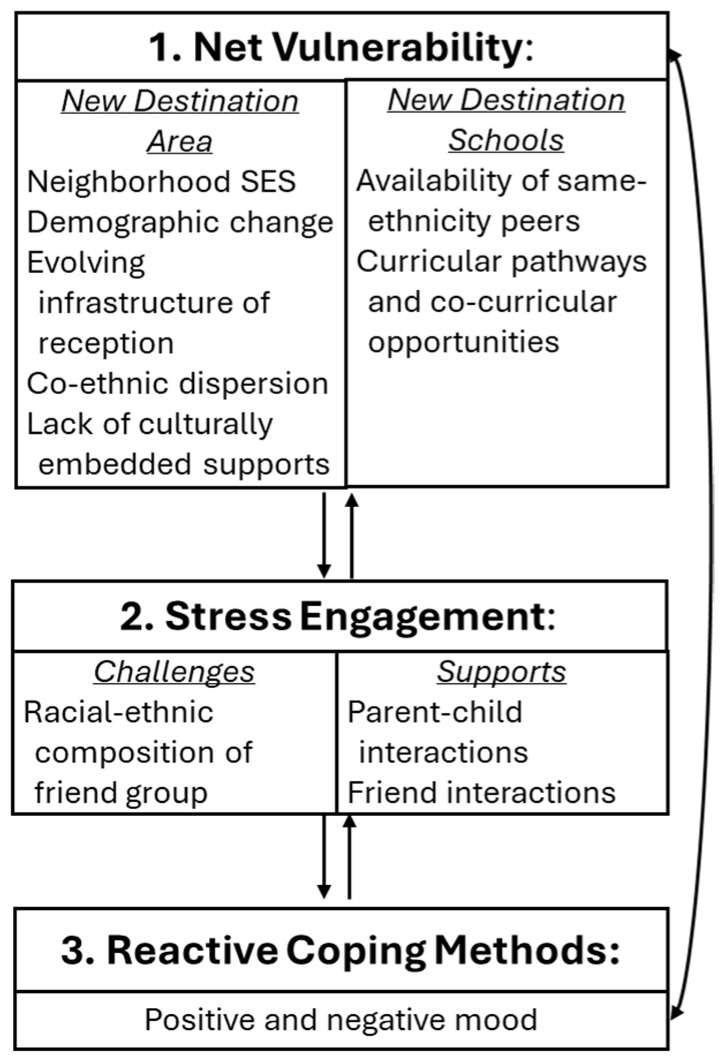
Conceptual model adapted from PVEST (Spencer et al., 1997).

In Figure 1, we provide a conceptual model for this study using the PVEST. Specifically, we conceptualize a new destination area as a broader contextual factor that may confer varying levels of risks or resources for Black and Latiné youth—relatively fewer co-ethnic peers for Latiné youth may be a risk whereas a greater proportion of co-ethnic peers for Black youth may be a resource. The overall neighborhood socioeconomic status (SES; low) may confer similar levels of risk (or stress; i.e., stress engagement) for both groups, which may shape youth’s daily peer and parent interactions. Next, the friend group’s racial–ethnic composition may act as a source of support or risk, thereby contributing to the adolescent’s level of stress (e.g., stress engagement). Central to the focus of this study, daily peer and parent–youth interactions may act as protective factors, yielding different levels of net vulnerability (i.e., these interactions coupled with the experience of the new destination area and friend group racial–ethnic composition) for each adolescent within each racial–ethnic group. Lastly, youth’s negative and positive mood are the varied immediate responses (i.e., reactive coping methods) to their context and interactions with others. This study provides an opportunity to explore normative, developmental experiences among Black and Latiné youth who experience challenges and opportunities that shape their everyday emotional well-being.

Friendships are important for adolescent well-being (Poulin & Chan, 2010; Vitaro et al., 2009) and two prominent perspectives guide this work (Vitaro et al., 2009). The social bonding perspective focuses on relationship quality, namely, the positive attributes of friendships and how they contribute to well-being and positive adjustment. The underlying mechanisms by which friendships impact youth developmental outcomes are feelings and a sense of belonging. The social interaction perspective also focuses on relationship quality and its impact on adjustment. However, the mechanism of focus is the quality of interactions between friends, with an emphasis on its negative attributes and their contributions to poor outcomes (e.g., depressive or anxiety symptoms). In the current study, following the social bonding perspective, we intentionally focus on positive interactions with friends to identify malleable, universal prevention targets that may enhance Black and Latiné youth’s emotional well-being.

Consistent with the idea that relational processes operate at both stable and fluctuating levels, recent work shows that between- and within-person variation in friendship dynamics are linked to adolescents’ internalizing symptoms (Schacter et al., 2023). Adolescents are likely to consider their relationships with individuals with whom they have frequent positive interactions to be warm, supportive, and close. In contrast, relationships characterized by a mix of positive and negative interactions may be characterized as unreliable and untrustworthy. Relatedly, positive everyday interactions with family members and peers may evoke positive emotion, fostering resilience in the face of other adversities. As a key component of resilience in the face of adversity, the buildup of positive emotion likely protects youth from the detrimental effects of stressful contextual experiences that could pose risks to emotional and behavioral health (Bai & Repetti, 2015). Ecological momentary assessments (EMAs) and daily diary methodology, in which adolescents report on thoughts, behaviors, and emotions every day over several days, are well-suited for capturing short-term resilience processes that have long-term implications for adolescent development. Moreover, by capturing “life as lived” and decreasing participant burden of recall, EMAs help to increase ecological validity (Bolger et al., 2003). With these advantages, the current study’s approach to assessing daily peer interactions and mood enriches the evidence base for PVEST (Spencer et al., 1997; Spencer, 2008).

### 1.2. New Destination Area

New destination areas are characterized by relatively recent demographic change and evolving infrastructures of reception, which may shape youth relational opportunities. More specifically, these areas are not ethnic enclaves where youth may access dense culturally embedded supports; these areas are often characterized by fewer co-ethnics and co-ethnic dispersion, thereby limiting resources that can shape the meaning and benefits of friendships (Seff et al., 2021). Emerging research in new destination contexts suggests that neighborhood conditions and racialized stressors remain salient predictors of adolescents’ adjustment, reinforcing the need for context-specific developmental evidence (Bermudez et al., 2025). In such settings, Latiné youth may be part of newer migration streams while Black youth may represent long-standing residents. However, both groups navigate shifting intergroup dynamics and peer opportunity structures that shape who is available to befriend and how safe or supportive cross-group contact feels. In schools within these contexts, peer opportunity structures may increase intergroup contact but simultaneously constrain access to culturally affirming support, depending on local social and school dynamics (Silver, 2015; Stanton-Salazar & Spina, 2005). In new-destination schools, peer opportunities are often structured through institutional pathways (e.g., extracurricular activities and clubs) that shape access to social capital, rather than emerging uniformly from demographic diversity alone (Silver, 2015), suggesting that even “available” cross-race or cross-ethnic friendships may differ in quality and emotional meaning. These contextual features may meaningfully condition whether everyday positive interactions with peers translate into emotional benefits. Despite growing attention to immigrant and newcomer adjustment, research on peers and emotional well-being relies on cross-sectional or multiwave designs and rarely tests same-day processes or the emotional implications of peer racial–ethnic composition in daily life (Russell & Gajos, 2020; Y. Wang, 2021). Accordingly, the present study examines daily positive friend interactions and mood among Black and Latiné adolescents in a new destination area, and tests whether these links vary by friend-group racial/ethnic composition and parent–adolescent interactions.

### 1.3. Interactions with Peers

Cross-sectional and longitudinal studies demonstrate the importance of peers for social and emotional development during adolescence. In Black and Latiné adolescents, peer relationship quality has often been examined alongside culture and stress. In Latiné youth, peer support reduced the strength of the association between discrimination and depression symptoms, and positively correlated with school belongingness, college attendance, and self-efficacy (Gonzalez et al., 2014). The protective effect of peer support is also observed in longitudinal studies (Benner et al., 2017; Cui et al., 2020; Quimby et al., 2018; M.-T. Wang et al., 2021). For example, peer support helped maintain social and emotional well-being in predominantly Latiné (85%) adolescents transitioning from eighth to ninth grade (Benner et al., 2017). Likewise, Black youth with more stable, positive peer interactions reported higher levels of self-esteem and school connectedness over three years (Quimby et al., 2018). In contrast, the absence of positive peer relationships is associated with difficulties in coping and poorer health in diverse adolescents (M.-T. Wang et al., 2021).

Studies of the daily associations between peer interactions and mood provide further support for the importance of peers (Bai et al., 2017; Chen et al., 2024; Chun et al., 2022; Griffin et al., 2019; Roshanaei et al., 2024; Weinstein et al., 2006). In a sample of ethnically diverse (45% White, 22% Latiné, 17.5% Black) 8- to 13-year-old youth, more school problems (i.e., academic and peer problems) were associated with greater negative mood on the given day (Bai et al., 2017). Another daily study of 10th graders (57% Latinx, 21% Biracial, 10% Asian, 9% White, 4% Black) found that peers’ racial teasing was associated with increased negative mood on the same day (Chen et al., 2024). Additionally, a daily diary study consisting of mostly Latiné adolescents (66%) who were temporarily living with friends or family due to loss of housing indicated that greater levels of peer support were associated with greater levels of positive mood on the same day (Griffin et al., 2019). Other daily diary studies conducted with a majority of White adolescent samples further suggest that friendship quality and positive peer interactions are associated with higher levels of happiness and positive mood, lower levels of anger, anxiety, and sadness, and lower variability in anger and anxiety (Herres et al., 2018; Mak et al., 2023). Despite the increase in sample diversity in studies of adolescent development in recent years, research on positive peer interactions has mostly been conducted in mostly White samples. Furthermore, although some studies have used daily diary methods to study peer processes in minorized youth (e.g., Douglass et al., 2016), emphasis on the environment beyond the school setting are uncommon. To our knowledge, only one study has explicitly compared Latiné youth in a new immigrant destination area to those in a historical receiving community (Potochnick et al., 2012). Using daily diary data, the authors found that, relative to youth living in a more established area (i.e., Los Angeles), Latiné youth in urban and rural North Carolina reported higher levels of daily happiness and daily depression symptoms; those in rural North Carolina also reported elevated anxiety. Importantly, youth in rural North Carolina experienced more discrimination, but they also reported more positive school climates, adult encouragement, and a greater percentage of days of positive ethnic treatment. Thus, youth in new destination contexts were exposed to both heightened discrimination and heightened social acceptance, with nativity differences partially accounting for disparities in depression and anxiety symptoms (Potochnick et al., 2012). However, this study focused exclusively on Latiné youth and examined ethnic treatment and school-based social climate, rather than daily positive peer interactions across diverse minoritized groups. Examining daily positive peer interactions among racially and ethnically diverse minoritized youth in new destination areas would extend this work by clarifying how normative peer processes operate alongside acculturation-related experiences to shape youths’ net vulnerability and resilience.

### 1.4. Racial–Ethnic Composition of the Friend Group

Peer or friendship homophily refers to the tendency for individuals to gravitate towards peers similar to themselves (Echols & Graham, 2013), with key sociodemographic characteristics (e.g., gender, race–ethnicity, educational level, religion) and/or values (e.g., similar educational orientation) as critical drivers of such similarities (McPherson et al., 2001). Adolescents’ friendships are frequently characterized by their racial–ethnic composition, such that youth are more likely to form close ties with peers who share their racial or ethnic background. At the same time, cross-racial and cross-ethnic friendships are common in diverse school settings and may carry distinct implications for emotional adjustment. A growing body of research suggests that the ethnic composition of adolescents’ friendship networks can shape socioemotional experiences (Witherspoon et al., 2023), particularly in contexts where race and ethnicity are salient features of daily life.

Several studies indicate that cross-ethnic friendships may confer emotional benefits under certain conditions. Cross-ethnic friendships have been associated with lower perceived vulnerability and, in some contexts, have buffered links between discrimination and adolescents’ socioemotional well-being; however, these effects vary by school climate and broader intergroup norms (Benner et al., 2018; Chan & Benner, 2025; Graham et al., 2014). Longitudinal research further suggests that reciprocated cross-ethnic friendships can predict declines in depressive symptoms over time for some youth (Kelleghan et al., 2019). Similarly, cross-race and cross-ethnic friendships have been linked to more favorable trajectories of psychological well-being, particularly in contexts characterized by limited same-ethnic representation or elevated prejudice (Liu et al., 2020). These findings suggest that friendship diversity may function as a resource for emotional adjustment when cross-group ties foster belonging and social integration.

Yet the emotional implications of cross-ethnic friendships are not uniformly positive. Research examining interracial best friendships during middle school indicates that associations with emotional well-being vary across racial and ethnic groups (McGill et al., 2012). Moreover, the benefits of cross-ethnic friendships appear contingent on broader intergroup climates; in supportive contexts they may promote well-being, whereas in less supportive climates they may not confer the same emotional advantages (Brenick et al., 2018).

At the same time, same-race or same-ethnic friendships provide stronger emotional support and social affirmation (Leszczensky et al., 2019), enhance school attachment and belonging (Rambaran et al., 2022; Ueno, 2009), and promote exploration of ethnic–racial identity (Lorenzo et al., 2024). Same-race friendships have also been uniquely linked to stronger ethnic–racial identity, private regard, and feelings of being understood, processes that may be especially salient for emotional validation in racially stratified contexts (Graham et al., 2014; Debrosse et al., 2025). Importantly, youth with exclusively interracial best friendships have reported lower emotional well-being than those with at least one same-race best friend, suggesting that the absence of same-race close ties may carry emotional costs for some adolescents (McGill et al., 2012). Together, these mixed findings underscore that friendship ethnic composition operates within systems of racialized power and social meaning; the racial–ethnic composition of friend groups may be experienced as a resource or risk.

Notably, studies examining friendship ethnic diversity and emotional adjustment often rely on between-person designs assessed at single or widely spaced time points. Far less is known about how adolescents’ day-to-day peer experiences unfold within friendship contexts that vary in racial and ethnic composition, particularly among Black and Latiné youth residing in new destination areas. To our knowledge, only one study has examined the racial or ethnic composition of friends on daily mood in a predominantly Black and Latiné youth sample. With college students, Debrosse et al. (2023) found that individuals who felt greater support in same-race close friendships experienced less depressive mood, and cross-race support was not associated with depressive mood (Debrosse et al., 2023). This set of findings suggests that in daily life, the mood benefits of positive interactions with friends may be especially potent when the friends have the same race or ethnicity as the adolescent. Consistent with PVEST, we examined the transactions between the broader peer context and the quality of specific peer interactions as important for shaping youth mood. Friendship composition and ethnic–racial identity appear to co-develop over time, with more diverse friendship networks predicting greater identity exploration and identity processes, in turn, shaping friendship patterns (Rivas-Drake et al., 2017; Kornienko et al., 2024). This information is especially meaningful for racial–ethnic minoritized adolescents living in new destination areas where opportunities for cross-race or cross-ethnic friendships are more plentiful for all adolescents and same-race or same-ethnic friendships are relatively minimal for newcomers (e.g., Latiné), but equally or moderately plentiful for established communities (i.e., Black).

### 1.5. Parent–Youth Interactions

Theoretical and empirical work underscore the salience that the quality of adolescents’ relationships with their parents has for youth emotional well-being, regardless of their race or ethnicity (Eugene, 2021; Mueller & Haines, 2012; Murry et al., 2009; Stafford et al., 2021). Accordingly, the quality of parent–youth interactions at the day level is also associated with youth mood. For example, in a sample of mostly White adolescents, family support was associated with better mood, and this relationship was stable from eighth to tenth grade (Weinstein et al., 2006). However, as with the research on peer interactions, Black and Latiné youth are relatively underrepresented in studies of parent–youth interactions in daily life.

Considered together, parent–adolescent relationship quality likely moderates same-day associations between adolescents’ peer interactions and mood. Researchers have proposed several theoretical models to explain how an adolescent’s relationships with their parents and peers improve their well-being (for a review, see Schacter & Margolin, 2019; Zhang et al., 2018). The Additive Model proposes that peer and parent support independently contribute to adolescent emotional well-being. The Reinforcement Model suggests that parent and peer support is interactive, as the benefits of support from one area are augmented by high levels of support from the other, with those who feel supported by both experiencing better well-being. The Toxic Friends Model, a variation in the reinforcement model, posits that a negative relationship with the parent puts the adolescent at risk for affiliating with friends whose support exacerbates their negative mood (Millgram et al., 2024). Finally, the Compensation Model states that parent or peer support may compensate for a lack of support from other individuals (Zhang et al., 2018).

A cross-cultural study of peer and parent support conducted on a sample of over 80,000 10–12-year-old youth from 32 countries found support for the reinforcement/toxic friends model. The level of individualism as opposed to collectivism at the country level, did not moderate this finding (Millgram et al., 2024). Within the U.S., cross-sectional and longitudinal studies with Latiné adolescents provide some evidence for the compensation model. For example, a study of Latiné adolescents in the ninth grade showed that adolescents’ affiliation with achievement-oriented peers was associated with their educational aspirations, even at low levels of parental support (Espinoza et al., 2014). The interplay between an adolescent’s relationships with their parents and peers likely differs across different time scales. For example, a daily diary study of perceived social support, happiness, and social connectedness in adolescents (32% identified as Hispanic or Latiné, 18% identified their race as Black) found evidence for a compensatory model of support at the between-person level and for an additive model of social support at the daily level (Schacter & Margolin, 2019). Few studies have investigated daily peer and parent–adolescent interactions together in Black and Latiné adolescents. However, minoritized youth and parents in new destination areas may espouse a broader spectrum of values and values alignment (Harris & Chen, 2023), necessitating a closer investigation of daily peer interactions in the context of parent–youth interactions.

### 1.6. Adolescent Positive and Negative Mood

Adolescence is a dynamic period of expansive emotional experiences. The breadth of emotions—both positive and negative—experienced during this period likely stems from biological, cognitive, and social changes—including peer changes (Hollenstein & Lougheed, 2013). For some individuals, these developments may yield more experiences of stress and more instances of reactive coping, a process that begins with a shift in mood states upon a stressful experience.

Broad definitions of coping encompass behavioral, emotional, and cognitive responses that manage one’s reactions to stress (Repetti & Bai, 2025). Youth’s negative and positive mood are the varied immediate responses (i.e., reactive coping methods) to everyday experiences, both stressful and protective. In studies involving daily diaries or EMAs, daily mood is a common outcome, shaped by interpersonal and ethnoracial factors such as violence exposure, racial discrimination, parental racial socialization, and general social support (Cheeks et al., 2020; Deane et al., 2021; Ortega-Williams et al., 2022). Daily diary research with diverse adolescents shows that social acceptance and discrimination, and other ethnoracially salient peer experiences can predict day-to-day fluctuations in psychological well-being (Potochnick et al., 2012; Y. Wang, 2021). Studies involving Latiné youth specifically have linked daily negative and positive mood to peer and academic stress, discrimination, cultural socialization practices and family relations (Del Toro et al., 2024; Santiago et al., 2017; Park et al., 2024; Potochnick et al., 2012; Valentino et al., 2025). However, prior studies have drawn on the experiences of youth who live in more segregated neighborhoods, wherein the majority of residents share the youth’s race or ethnicity, or have explored more established destination communities with greater racial–ethnic diversity. Furthermore, studies that examine peers and families as intersecting sources of support are limited—an important research direction to enrich strength-based perspectives of adolescent development.

### 1.7. Current Study

Informed by the PVEST (Spencer et al., 1997; Spencer, 2008), this study aimed to examine the benefits of supportive, understanding, and complimentary experiences with friends—hereafter referred to as positive friend interactions—on the daily mood of Black and Latiné adolescents. We investigated how same-day associations between positive friend interactions and mood vary across two contextual factors: the racial–ethnic composition of an adolescent’s friend group and the quality of parent–adolescent interactions. Using data from two pilot daily diary studies—one with Black and one with Latiné adolescents living in a new destination area—our first aim examined the same-day association between daily positive friend interactions and both negative and positive mood. Consistent with prior research on the daily associations between peer interactions and mood (Bai et al., 2017; Chen et al., 2024; Chun et al., 2022; Griffin et al., 2019; Roshanaei et al., 2024; Weinstein et al., 2006), we hypothesized that, on days when adolescents have positive friend interactions, they would report greater positive mood and lower negative mood. For the second aim, we examined at what levels of friend racial–ethnic homophily, the same-day association between positive friend interactions and mood would be the strongest. We hypothesized that for Black and Latiné adolescents, the same-day association would be the strongest for youth with more same-race or ethnicity friends (Debrosse et al., 2023). For the third aim, we tested the same-day association between positive friend interactions and mood, as moderated by parent–youth interactions. We predicted that the same-day association would be strongest on days when adolescents reported less positive parent–youth interactions and for adolescents who reported low levels of positive parent–youth interactions across the daily diary period consistent with the compensatory model of peer and parent support (Zhang et al., 2018). Importantly, although daily diary/EMA methods are increasingly used in developmental research, prior work rarely tests the same-day links between peer experiences and mood while also attending to the racial–ethnic composition of friend groups (Russell & Gajos, 2020; Y. Wang, 2021). To our knowledge, no prior study has examined same-day associations between positive friend interactions and daily mood among both Black and Latiné adolescents residing in a new destination area, while simultaneously testing whether these links vary by friend-group racial–ethnic composition and by parent–adolescent interaction quality. This design clarifies whether positive peer processes operate as immediate, strength-based resources under distinct contextual constraints.

## 2. Method

### 2.1. Participants

The participants in this project were part of two mixed-method pilot studies conducted in 2018 and 2019–2023. Both studies applied intensive longitudinal methods to examine sociocontextual factors that shape the development of adolescents and assessed the acceptability of data collection procedures with qualitative methods (e.g., focus groups). The first pilot study included 21 Latiné adolescents and their parents residing in a small Northeastern U.S. city where Latiné residents accounted for 24.7% of the population (U.S. Census Bureau, n.d.). Participants were recruited through community events, flyers, and snowball recruitment techniques. To participate in this study, participants had to identify as Latiné, be 11–14 years of age, read English and/or Spanish at a third-grade level, and reside with the participating parent. The second pilot study included 36 Black adolescent–parent dyads living in the same city as participants from the first pilot study, where 51.2% of the population was Black at the time of the study (U.S. Census Bureau, n.d.). Similarly, participants were recruited through flyers, community events, and through snowball recruitment. To be part of this study, participants had to identify as Black/African American, be between the ages of 11 and 17, understand and read English at a third-grade level, and reside with the participating parent.

The Latiné participants’ mean age was 12.76 (*SD* = 1), and there was an almost equal distribution of boys (*n* = 10; 47.6%) and girls (*n* = 11; 52.4%). Half of the Latiné participants ethnically identified as Puerto Rican (57.10%) and were born in the U.S. mainland (52.40%). The mean age of Black participants was 13.65 (*SD* = 2.29). In terms of gender, 52.9% identified as female, 44.1% identified as male, and 2.9% identified as neither male or nor female, which we described as gender diverse. More information about both samples is available in Table 1.

### 2.2. Procedure

Participants in both studies completed a baseline survey that collected data on demographics, family and peer relationships, school and neighborhood experiences, and well-being. After the baseline survey, participants received smartphones and instructions on using them for the ecological momentary assessment (EMA) portion of the study, which further assessed their relationships, mood, and physical and psychological well-being. Latiné youth in the first pilot study completed three EMA surveys over a 14-day period. Black youth in the second pilot study completed five EMA surveys over a 21-day period. After completing the EMA portion of the study, some participants participated in focus-group interviews in which they shared their experiences using the smartphone and completing the EMA surveys. Black youth in the second pilot study also completed a 3-month follow-up survey. For consistency across the two samples, the current study uses adolescent data from the baseline survey and the first 2 weeks of EMAs. We used data from the afternoon and before-bed EMAs (2 surveys per day) from only the 10 weekdays, as adolescents are more likely to consistently interact with peers on weekdays. Of the 57 adolescents who completed at least 1 EMA survey, the average participant completed 74.65% (*SD* = 26.66%; *Range* = 0–100%) of the 20 possible surveys (afternoon and before bed) across 10 days. The two samples did not differ in compliance rates across the 10 days (*t*(55) = 1.37, *p* = 0.18).

### 2.3. Measures

#### 2.3.1. Racial–Ethnic Composition of Friend Groups

In the baseline survey, participants listed up to three close friends and reported the race or ethnicity of each friend. For each friend, a code of 0 was assigned if the friend was part of a different racial or pan-ethnic (e.g., Latiné) group than the participant. A code of 1 was assigned if the friend was from the same racial or pan-ethnic group as the participant (e.g., Puerto-Rican participant and their Dominican friend would be coded as sharing the same pan-ethnicity). Friend homophily was computed as the proportion of same- same-race/pan-ethnic close friends by dividing the number of same-race/pan-ethnic friends by three (the maximum possible number of friends listed). Of the 49 adolescents who provided this information, 3 reported 0%, 8 reported 33%, 13 reported 67%, and 25 reported 100% same-race–ethnicity friends.

#### 2.3.2. Daily Positive Friend Interactions

On the afternoon EMA surveys, adolescents responded to 3 items about interactions with friends at school on a 4-point scale (1 = *Not at all*, 4 = *Very much*); item scores were averaged (Griffin et al., 2019). Positive experiences included items about support, understanding, and praise (e.g., “did any friends give you emotional or social support”). The average α over the 10 days was 0.92 (*SD* = 0.07, *Range* = 0.78–0.99).

#### 2.3.3. Daily Positive Interactions with Parents

At the end of the day, adolescents completed 4 items about positive interactions with their parents (e.g., “listened”) (Griffin et al., 2019). Item scores ranged from 1 (*Not at all*) to 4 (*Very much*) and were averaged. On average, α was 0.92 (*SD* = 0.02, *Range* = 0.88–0.94).

#### 2.3.4. End of Day Negative and Positive Mood

The end-of-day EMA surveys assessed adolescents’ positive and negative mood using an adapted 12-item scale (Griffin et al., 2019). Participants reported on how much they felt each of 5 positive mood items (e.g., happy) and 7 negative mood items (e.g., sad) since the last assessment on an 11- or 10-point response scale. Due to discrepancies in response scales between the two pilot studies, responses on the 11-point response scale were recoded to a 10-point scale (1 = *Not at all*, 10 = *Very much*), and we averaged the items. Mean α were 0.82 (*SD* = 0.04, *Range* = 0.74–0.86) for positive and 0.68 (*SD* = 0.12, *Range* = 0.53–0.86) for negative mood.

#### 2.3.5. Covariates

The included covariates were gender (0 = male, 1 = female; 46.3% male) and ethnicity (0 = Black, 1 = Latiné; 63.2% Black).

### 2.4. Analysis Plan

We calculated descriptive statistics in SPSS version 29.0.0.0 (IBM Corp., 2022). To accommodate the nested structure of the data wherein days were nested within adolescents, we conducted mixed linear models using the Mixed Procedure in SAS software version 9.4 (SAS Institute Inc., 2025 (Cary, NC, USA)), with maximum likelihood estimation (Curran & Bauer, 2011). Predictors that varied by day (i.e., positive friend interactions) were decomposed into two components via grand-mean (i.e., a person’s average) and person-mean centering (i.e., a score for a day relative to the person’s average, daily level). Random intercepts were estimated in the models. The inclusion of random slopes led to overfitting, and thus were not included in the reported models.

To address our first aim, we examined the associations between positive friend interactions and positive and negative mood. Average and daily levels of positive friend interactions were key independent variables of interest, and positive and negative mood, dependent variables. To address our second aim of testing the racial–ethnic composition of friends (i.e., friend homophily) as a moderator, we expanded our model to include friend homophily and the interaction term between friend homophily and daily positive friend interactions. For the third aim of testing positive parent interactions as moderators, we added person-mean-centered and grand mean levels of positive parent interactions, as well as their interactions with daily positive friend interactions. We probed the simple slopes of significant interaction terms. Each model was tested on the combined total sample and separately for Black and Latiné youth. All models included gender as a covariate. The combined total sample models also included race–ethnicity as a covariate.

Out of the 57 participants, one participant did not complete any eligible daily diaries, two participants did not provide an age or gender identity and one participant was dropped from the analysis due to our binarization of the gender variable. Thus, the total analytical sample for Aim 1 was 53 participants (33 Black youth and 20 Latiné youth; n_obs_ = 248). An additional 6 participants did not report on the race–ethnicity of their friend, reducing the analytical sample for Aim 2 to 47 (27 Black youth and 20 Latiné youth; n_obs_ = 226). Last, the analytical sample for Aim 3 was 49 youth (29 Black and 20 Latiné youth; n_obs_ = 248), as four youth had no days with complete data on their interactions with friends and parents. Little’s test conducted on demographic variables and homophily indicated that these data were missing completely at random (MCAR; X^2^ = 20.06, *df* = 13, *p* = 0.094).^1^

## 3. Results

### 3.1. Descriptive Statistics

Table 2 displays descriptive statistics of key study variables (averaged for each person) for the total, Black and Latiné samples, as well as the results of independent sample *t*-tests. Independent-sample *t*-tests indicated that Black and Latiné youth did not significantly differ on study variables. Table 3 displays the correlations between key variables at the between-person level of analysis for the total sample. Adolescents who reported greater levels of positive friend and positive parent interactions also reported more positive and less negative mood. In addition, positive friend and parent interactions were correlated.

### 3.2. Associations Between Positive Friend Interactions and Mood

Results of models testing the same-day association between positive friend interactions (i.e., independent variable) and positive and negative mood (i.e., dependent variables) are presented in Table 4 and Table 5.

#### 3.2.1. Total Sample

Having positive interactions with friends was not associated with youths’ positive or negative mood (*B* = 0.05, *SE* = 0.16, *p* = 0.743; *B* = −0.04, *SE* = 0.12, *p* = 0.730).

#### 3.2.2. Black Youth

Black youth who reported greater mean levels of positive interactions with friends reported more positive mood during the daily diary period (*B* = 0.69, *SE* = 0.38, *p* = 0.078). However, this association was not observed at the daily level for positive or negative mood (*B* = −0.02, *SE* = 0.19, *p* = 0.911; *B* = −0.06, *SE* = 0.15, *p* = 0.721).

#### 3.2.3. Latiné Youth

Having positive interactions with friends was not associated with Latiné youths’ positive or negative mood (*B* = 0.12, *SE* = 0.24, *p* = 0.614; *B* = −0.03, *SE* = 0.17, *p* = 0.873).

### 3.3. The Proportion of Same-Race or Ethnicity Friend as a Moderator

Next, we added friend homophily, or the proportion of close friends who share the same race–ethnicity as a moderator of the same-day association between positive friend interactions and mood.

#### 3.3.1. Total Sample

Friend homophily did not moderate the association between positive friend interaction and positive mood (*B* = −0.05, *SE* = 0.60, *p* = 0.935) or negative mood (*B* = −0.12, *SE* = 0.45, *p* = 0.800).

#### 3.3.2. Black Youth

**Figure 2:**
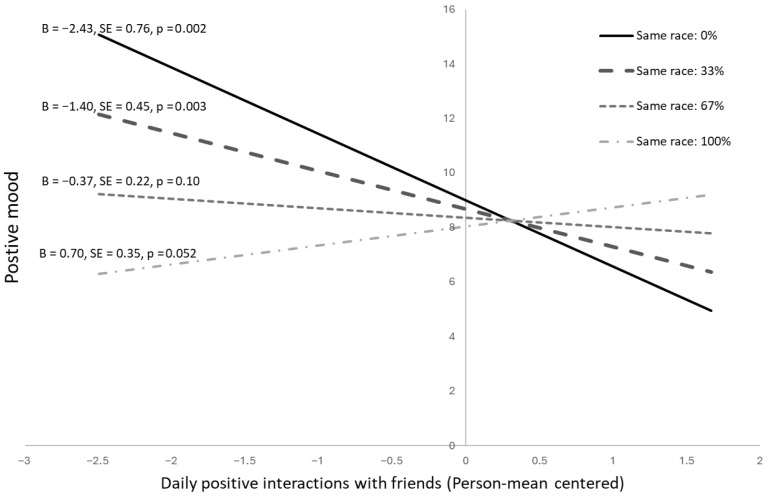
Same-day association between positive friend interactions and positive mood, moderated by the racial/ethnic composition of close friends in Black adolescents.

We found a significant interaction between friend homophily and daily positive friend experiences in predicting positive mood (*B* = 3.13, *SE* = 1.01, *p* = 0.003; see Table 4 and Figure 2). For Black youth with none or few same-race friends, the association between positive friend interactions and positive mood was negative. When youth reported more positive friend interactions, they reported lower positive mood (0 same-race friends: *B* = −2.43, *SE* = 0.76, *p* = 0.002; one same-race friend: *B* = −1.40, *SE* = 0.45, *p* = 0.003). For youth with many same-race friends, the association between positive friend interactions and positive mood was positive (3 same-race friends: *B* = 0.70, *SE* = 0.35, *p* = 0.052). When youth reported more positive friend interactions, they reported more positive mood at the end of the day. Friend homophily did not interact with daily positive friend interactions in the prediction of negative mood (*B* = 0.46, *SE* = 0.89, *p* = 0.606).

#### 3.3.3. Latiné Youth

For Latiné youth, the proportion of same-ethnicity friends did not significantly moderate the same-day association between and daily positive friend interactions and positive mood (*B* = −0.88, *SE* = 0.76, *p* = 0.250) or negative mood (*B* = −0.27, *SE* = 0.55, *p* = 0.617).

### 3.4. Moderation by Positive Parent Interactions

As with friend homophily, we examined positive parent interactions—both at mean and daily levels—as moderators of the association between positive friend interactions and mood.

#### 3.4.1. Total Sample

Mean or daily levels of positive parent interactions did not moderate the same-day association between positive friend interactions and positive mood (*B* = −0.11, *SE* = 0.16, *p* = 0.501; *B* = −0.21, *SE* = 0.31, *p* = 0.490, respectively). Additionally, mean and daily levels of positive parent interactions did not significantly interact with daily levels of positive friend interactions in the prediction of negative mood (*B* = −0.19, *SE* = 0.13, *p* = 0.141; *B* = 0.26, *SE* = 0.25, *p* = 0.289, respectively). Notably, average and daily levels of positive parent interactions were positively associated with positive (*B* = 1.34, *SE* = 0.23, *p* ≤ 0.001; *B* = 0.80, *SE* = 0.15, *p* ≤ 0.001, respectively) and negative mood (*B* = −0.42, *SE* = 0.20, *p* = 0.041; *B* = −0.29, *SE* = 0.12, *p* = 0.015, respectively).

#### 3.4.2. Black Youth

**Figure 3:**
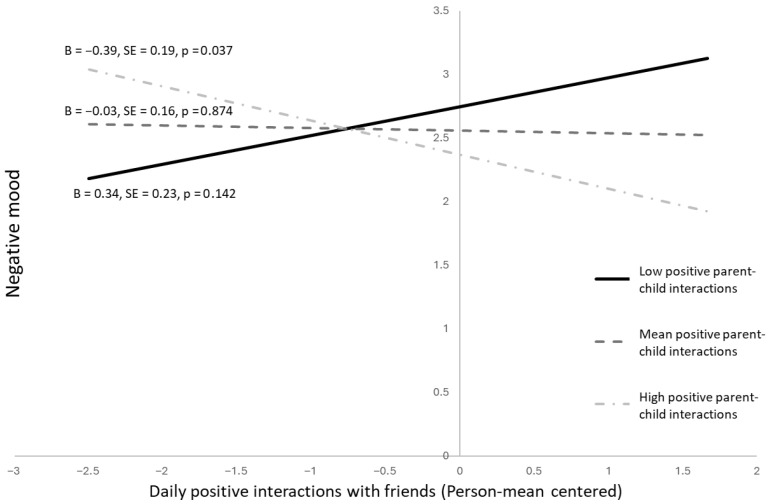
Same-day association between positive interactions with friends and negative mood, moderated by the mean levels of positive interactions with parents in Black adolescents.

Mean or daily levels of positive parent interactions did not moderate the same-day association between positive friend interactions and positive mood for Black youth (*B* = 0.02, *SE* = 0.18, *p* = 0.917; *B* = 0.71, *SE* = 0.44, *p* = 0.112, respectively). However, mean levels of positive parent interactions interacted with daily levels of positive friend interactions in the prediction of negative mood (*B* = −0.41, *SE* = 0.15, *p* = 0.009). Among Black youth with higher mean levels of positive parent interactions, the same-day association between positive friend interactions and negative mood was negative (*B* = −0.39, *SE* = 0.19, *p* = 0.037). When youth reported more positive friend interactions, they reported lower negative mood. This relation was not observed for those with average (*B* = −0.03, *SE* = 0.16, *p* = 0.874) or below average (*B* = 0.34, *SE* = 0.23, *p* = 0.142) levels of positive parent interactions. See Figure 3. Average and daily levels of positive parent interactions were associated with positive (*B* = 1.07, *SE* = 0.30, *p* ≤ 0.001; *B* = 0.91, *SE* = 0.20, *p* ≤ 0.001, respectively), but not negative (*B* = −0.32, *SE* = 0.28, *p* = 0.272; *B* = −0.09, *SE* = 0.18, *p* = 0.617, respectively) mood.

#### 3.4.3. Latiné Youth

Mean or daily levels of positive parent interactions did not moderate the same-day association between positive friend interactions and positive mood (*B* = −0.22, *SE* = 0.30, *p* = 0.465; *B* = −0.63, *SE* = 0.44, *p* = 0.159, respectively) or negative mood (*B* = 0.14, *SE* = 0.22, *p* = 0.527; *B* = 0.10, *SE* = 0.32, *p* = 0.762, respectively) for Latiné youth. However, average and daily levels of positive parent interactions were associated with more positive (*B* = 1.71, *SE* = 0.35, *p* ≤ 0.001; *B* = 0.76, *SE* = 0.21, *p* ≤ 0.001, respectively) and lower negative mood (*B* = −0.73, *SE* = 0.24, *p* = 0.003; *B* = −0.39, *SE* = 0.15, *p* = 0.010, respectively).

## 4. Discussion

Interactions with friends and parents are important for adolescents’ well-being. Although some scholarship about how daily experiences with peers and parents shape youth’s daily mood exists, there is a dearth of literature that examines normative developmental experiences with racial-ethnically minoritized youth (Padilla et al., 2024). Grounded in cultural-contextual developmental theories (e.g., PVEST; Spencer et al., 1997), the current study examined the benefits of positive friend interactions for the daily mood of Black and Latiné youth in a new destination area. We were especially interested in understanding the degree to which friend compositional factors (i.e., proportion of same-race–ethnicity friends) and parent–adolescent interactions might moderate the link between positive friend interactions and adolescent positive and negative mood. In general, at the daily level, having more positive friend interactions was unrelated to mood, positive or negative. However, contextual characteristics mattered for Black youth. Black youth seemed to be the most vulnerable or sensitive to context, such that youth who had mostly non-Black friends (i.e., greater friend group diversity; more cross-race or cross-ethnic friends) reported *lower* positive mood on days when they had greater than usual levels of positive friend interactions. Among Black youth who had, on average, more positive interactions with their parents, greater levels of daily positive friend interactions were associated with lower negative mood. These patterns are especially noteworthy when considering the new destination area where Black individuals are the numerical majority. This pattern may signal the lingering effects of structural racism and Black youth developing in an anti-Black society (Murry, 2022; Neblett & Neal, 2022), where stereotypes and discrimination continue as well as the importance of the family environment. Such a duality demonstrates the concept of net vulnerability (Spencer, 2008), highlighting how challenges and supports may operate within and across key developmental contexts. Overall, our results provide a complex picture of the contextualized associations between positive friend interactions and the youth’s mood, but only for Black/African American youth. Results for Latiné youth were not significant. Below, we discuss our findings in relation to the extant literature.

### 4.1. The Effects of Positive Friend Interactions

Following the social bonding perspective (Vitaro et al., 2009), we expected that positive friend interactions would be associated with adolescent well-being and positive adjustment. Contrary to our hypotheses, positive friend interactions were not significantly associated with mood at the daily level. In daily studies with predominantly White samples, scholars often demonstrate that positive friend interactions are associated with more positive mood (e.g., happiness) and less negative mood (e.g., sadness, anger) (Herres et al., 2018; Mak et al., 2023). In comparison, daily studies with more diverse populations (though few) tend to focus on negative friend interactions or peer problems and find that such problematic interactions are associated with more negative mood (Bai et al., 2017; Chen et al., 2024). We intentionally focused on positive friend interactions as part of the normative developmental experiences of Black and Latiné youth, homing in on the potential for these interactions to create a sense of belonging, connection, and safety. However, in this sample of Black and Latiné youth in a new destination area, engaging positively with friends did not confer such benefits for mood until we examined these associations in the context of friend compositional factors and parent–youth relationship quality, particularly for the Black youth sample.

Unexpectedly, for the Latiné sample, no significant associations were found when examining the role of friend support on daily mood. Only the support from parents was associated with greater positive and lower negative mood in our sample. There is some prior evidence that when examining the contributing role of parents, siblings, and peers/friends on Latine youth’s wellbeing, significant findings are more consistently found for the contribution of family members (Padilla et al., 2024). Additionally, it is possible that peer support is more consistently related to individual-level factors that fluctuate less across days such as sense of belonging, or may differ by other important variables like developmental period (e.g., early vs. late adolescence). For example, a cross-sectional study with young Latiné females of Mexican origin revealed that friend support predicted higher levels of self-esteem for older adolescents but for younger girls, more friend support predicted lower self-esteem (Bámaca-Colbert et al., 2017). Given the limited existent research on the role of peers and friends on the wellbeing of Latiné youth (Padilla et al., 2024), more research is necessary to make major conclusions.

### 4.2. Contextualized Effects of Positive Friend Interactions

For Black and Latiné youth who are developing in a stratified society that may devalue them and in contexts where anti-Black racism and xenophobia are ever-present, experiences of discrimination (Benner et al., 2018; Chen et al., 2024; Niwa et al., 2014), othering (Brietzke & Perreira, 2017; Sellers et al., 2006; Silver, 2015), or threats to contextual belonging (Delgado et al., 2016; Potochnick et al., 2012; Rambaran et al., 2022) may be more common, making it critically necessary to identify the contextual characteristics that may support (or undermine) positive experiences in key developmental settings (i.e., peer and parent). Therefore, we explored interactive associations in contexts where positive friend interactions may be beneficial, considering the racial–ethnic composition of friends, as well as how supportive parent–adolescent interactions may compensate for less positive interactions in other domains.

#### 4.2.1. Racial–Ethnic Composition of the Friend Group

Being around others who are like you on some identity dimension is theorized to increase feelings of safety, belonging, and connection. Many peer scholars have examined peer group composition factors, such as friend homophily—the proportion of friends who share the same individual characteristics as the target adolescent—and found that same-race friendships are associated with greater emotional well-being and racial–ethnic identity (Leszczensky et al., 2019; Lorenzo et al., 2024). However, many of these studies rely on cross-sectional or multi-wave designs and focus on identity or school adjustment rather than daily mood, leaving open questions about how friend group racial composition shapes momentary emotional experiences. At the same time, cross-race friendships have been linked with positive psychological well-being (Karataş et al., 2021). However, most of these studies have not specifically focused on mood or daily experiences.

We hypothesized that the racial–ethnic composition of Black and Latiné adolescents’ peer groups would impact the daily association between positive friend interactions and positive and negative mood, such that having more same-race friends would amplify the benefits of positive friend interactions on mood. Consistent with this hypothesis, Black youth in our sample with one or no Black friends (i.e., greater racial–ethnic diversity in friend group; fewer same-race friends) reported lower positive mood on days with more positive friend interactions. This finding suggests that these Black youth are not getting the boost or benefit of having daily positive peer interactions, when the majority of their friends do not share the same race. Black youth who report being a racial minority member among close friends may be finding seemingly supportive messages from cross-race friends unhelpful and potentially hurtful, whatever the messenger’s intent may have been. It is also possible that friends are trying to support youth on days that the youth finds difficult or stressful for a myriad of reasons. With the scope of the current study, we are unable to tease apart these plausible explanations

This finding is somewhat similar to Debrosse et al. (2023), who found that in Black and Latiné college students, more support from cross-race friends was not associated with depressive mood (Debrosse et al., 2023); only support from same-race friends was associated with lower depressive mood. However, why do positive interactions with cross-race friends not improve the daily moods of Black youth? Perhaps the daily realities of Black youth’s experiences are not captured by a compositional measure alone. Additional information about the content of conversations and what is discussed is necessary to describe the impact of positive friend interactions on mood. If the content of conversations is related to bias, discrimination, oppression, or other stressors, supportive positive interactions with peers who are not of the same race–ethnicity may not compensate for the intensity of the ubiquity of anti-Black sentiment in the United States. Further, Black youth’s own racial–ethnic identity development and understanding of themselves in relation to the world may shape how they interpret and internalize interactions with cross- and same-race friends, contributing to identity validation and enactment (Rivas-Drake et al., 2017; Santos et al., 2017; Seaton et al., 2017). For example, how Black youth think about Black people (private regard) and how Black youth think others view Black people (public regard) may color how Black youth make meaning of their interactions—whether positive or negative—with their peers (Rivas-Drake et al., 2017; Santos et al., 2017). Relatedly, Black youth’s racial centrality (i.e., importance of being Black to one’s self-concept) may increase positive friend interactions (Hoffman et al., 2019) but also highlight differences and bias in cross-race friendships. Each of these plausible explanations warrants further investigation and elucidates the complex and dynamic nature of adolescent-peer relationships.

#### 4.2.2. Supportive Parent–Adolescent Interactions

Positive interactions with parents promoted positive mood and reduced negative mood on average and at the daily level. Youth who, on average, had higher levels of positive interactions with parents reported higher levels of positive mood and lower levels of negative mood during the study. In addition, on days when youth reported higher levels of positive interactions with parents than their own average, they felt more positive and less negative mood. This finding is consistent with prior research on the enduring and short-term benefits of positive parent–youth interactions on adolescent emotional well-being (Bai & Repetti, 2015; Yap et al., 2014). The finding that positive parent–youth interactions are associated with improved mood at bedtime, whereas positive friend interactions did not have a significant main effect on mood, may also reflect a time effect. On school days, adolescents are more likely to interact with peers at school and parents at the end of the day; thus, the latter may have a stronger association with youth mood at bedtime.

Consistent with the reinforcement model rather than the compensation model (Zhang et al., 2018), we found that Black adolescents who, on average, endorsed high levels of positive parent–youth interactions showed lower negative mood on days when they had more positive interactions with friends. Positive interactions with friends only seem to be beneficial to Black youth who consistently report positive interactions with parents or caregivers. For Latiné youth, positive interactions with parents were consistently associated with better mood, regardless of the quality of their interactions with friends. This indicates that for Latiné youth, the quality of the parent–youth relationship remains most critical to their emotional well-being, even during adolescence when peer relations become more salient. Although the average Latiné youth reported having mostly same-ethnicity friends, the youth in our sample lived in a new destination area where Latiné residents accounted for 24.7% of the population. Thus, the significance of parent–youth interactions noted in our study may be, in part due to their minoritized status and/or their espousal of family-oriented values (Hernández & Bámaca-Colbert, 2016).

### 4.3. Limitations

Our study findings must be interpreted with recognition of several limitations. First, our daily surveys did not assess whether friend interactions occurred in person or online. As the questions about friend interactions were administered in the afternoon, rather than at the end of the day, we assume they were more likely to take place in person at school. However, adolescents often connect with peers their age online, and the potential benefits of online connections amid risks must be further investigated (Maxie-Moreman & Tynes, 2022; Nesi et al., 2018). Relatedly, our measure of homophily is a proxy measure of the race–ethnicity of friends who the participants interacted with on a given day of reporting. Friends referenced in the daily surveys are not necessarily the same three friends referenced in the baseline survey. Second, our Black and Latiné samples are pan-ethnic; most of the Black youth were African American, and the majority of the Latiné youth reported that they were Puerto Rican. Our focus on pan-ethnic groups limits our ability to examine differences by ethnicity, culture, heritage, and visible “race.” However, youth may be sensitive to cultural or visible differences, and investigations of intercultural friendships may deepen our understanding of peer relations among adolescents. Also, there are likely other important cultural process variables that should be included in these analyses to get a more complete picture of these developmental processes (Coll et al., 1996; Spencer et al., 1997). For example, racial–ethnic identity (Seaton et al., 2017), racial–ethnic socialization (Y. Wang & Lin, 2023), discrimination experiences (Chen et al., 2024), and acculturative stress (Wasserman et al., 2021) are cultural assets and stressors that may explain or modify the links between positive friend interactions and the youth’s mood.

Furthermore, youth were recruited from a new destination area. Findings may not generalize to adolescents in regions with more established communities of immigrant families or communities of ethnicities that are not represented in our sample. Our small sample size precludes a detailed examination of additional demographic moderators, which can increase our understanding of how our findings may generalize. Finally, although our survey procedures were designed to minimize recall bias and burden, they may also introduce limitations. Adolescents are likely to interact with peers during the day and parents at the end of the day. As a result, the association between interactions with parents and mood at the end of the day may be biased by shared method variance. Further research with more frequent assessments of positive and negative mood may address this and compare the effects of peer versus parent–youth interactions on youth mood.

## 5. Conclusions

Despite these limitations, the current study extends our understanding of normative social and emotional development in Black and Latiné adolescents living in new destination areas, supporting more inclusive theorizing (Spencer, 2008). Positive interactions with friends were associated with improved mood for Black adolescents who endorsed high levels of support from their own parents, but with poorer mood for Black adolescents who had mostly non-Black friends. Together, our study shows how short-term everyday experiences with peers may contribute to Black and Latiné youth’s emotional well-being. Investigations of contextual vulnerabilities and assets are important to understanding the social and emotional development of racially or ethnically minoritized youth.

## Figures and Tables

**Table 1:** Adolescents’ demographic information (*N* = 57).

	Black/African American (*N* = 36)	Latiné (*N* = 21)
**Age (years)**	*M* = 13.18, *SD* = 2.29, *Range* = 11–17	*M* = 12.76, *SD* = 1.00, *Range* = 11–14
**Gender**		
Female	52.90%	52.40%
Male	44.10%	47.60%
Gender-diverse	2.90%	
**Birth Country**		
U.S. Mainland	97.10%	52.40%
Puerto Rico	-	42.90%
Dominican Republic	-	4.80%
Jamaica	2.90%	-
**Self-described Race**		
Black	91.20%	
Black/White	2.90%	
Black/Asian	2.90%	
White	-	
**Self-described Ethnicity**		
Puerto Rican	-	57.10%
Mexican	-	9.50%
Dominican	-	4.80%
Guatemalan	-	4.80%
Puerto Rican/White	-	4.80%
Dominican/Black	-	4.80%
Peruvian	-	4.80%
Other	2.90%	9.60%
**Parent**		
Biological Mother	83.30%	95.20%
Biological Father	11.10%	4.80%
Other	5.60%	-
**Parent Education**		
Less than high school	2.80%	38.10%
High school	5.60%	14.30%
Some college or associate degree	52.70%	
Bachelor’s degree or more	39.00%	9.50%
**Parent Marital Status**		
Not married or cohabitating	47.00%	28.60%
Married/cohabitating	36.10%	47.60%
Widowed	5.60%	-
Separated	2.80%	14.30%
Divorced	8.30%	9.50%

**Table 2:** Descriptive statistics.

Variable	Total (*N* = 56)			Latiné (*N* = 20)	Black (*N* = 36)	
	*N*	*M* (*SD*)	Range	*M* (*SD*)	*M* (*SD*)	*t* (*df*)
Average Negative Mood	56	2.13 (1.07)	1–5.98	2.04 (1.09)	2.19 (1.07)	−0.49 (54)
Average Positive Mood	56	7.28 (1.92)	3.4–10	7.46 (1.85)	7.18 (1.97)	0.51 (54)
Average Positive Friend	56	1.73 (0.83)	1–4	1.95 (0.86)	1.61 (0.80)	1.70 (54)
Average Positive Parent	56	2.72 (0.90)	1.08–4	2.68 (0.93)	2.73 (0.89)	−0.21 (54)
Proportion Same-Race/ethnicity Friends	48	0.74 (0.32)	0–1	0.73 (0.35)	0.75 (0.3)	−0.18 (46)
Gender	54	0.55 (0.50)	0–1	0.55 (0.51)	0.55 (0.51)	0.03 (51)
Age	53	13.33 (2.04)	10–18	12.8 (1.01)	13.65 (2.23)	−1.60 (52)
Compliance Rate	56	0.84 (0.22)	0.2–1	0.91 (0.18)	0.81 (0.23)	1.74 (54)

Notes. TIES = 0; PARADE = 1. Compliance = number of completed study days divided by 10.

**Table 3:** Bivariate correlations between youth gender, age, proportion of same-race friends, and mean levels of key independent and dependent daily diary variables in the combined sample (Max *N* = 57).

		1	2	3	4	5	6	7	ICC
1	Negative mood	--							0.49
2	Positive mood	−0.40 **	--						0.62
3	Positive friend interactions	−0.10 *	0.44 *	--					0.65
4	Positive parent interactions	−0.28 **	0.62 **	0.56 **	--				0.65
5	% of same-race–ethnicity friends	−0.19 **	−0.11 *	−0.06	0.04	--			--
6	Gender (% female)	0.06	−0.19 **	0.09	0.13 *	0.12 *	--		--
7	Age	0.02	−0.16 **	−0.24 **	−0.20 **	0.15 **	−0.02	--	--

** *p* < 0.01, * *p* < 0.05.

**Table 4:** Mixed linear effects models examining associations between positive friend interactions and positive mood.

	Positive Friend Interactions			Positive Friend Interactions × Homophily			Positive Friend Interactions × Positive Parent Interactions		
	Total	Black	Latiné	Total	Black	Latiné	Total	Black	Latiné
	*B* (*SE*)	*B* (*SE*)	*B* (*SE*)	*B* (*SE*)	*B* (*SE*)	*B* (*SE*)	*B* (*SE*)	*B* (*SE*)	*B* (*SE*)
Intercept	7.87 (0.44) ***	8.02 (0.46) ***	7.64 (0.64) ***	8.42 (0.81) ***	8.99 (0.98) ***	7.82 (1.09) ***	7.79 (0.34) ***	7.88 (0.38) ***	7.88 (0.43) ***
Avg. Friend	0.44 (0.29)	0.69 (0.38) ’	0.21 (0.43)	0.55 (0.29) ’	0.95 (0.38) *	0.22 (0.43)	0.12 (0.15)	0.11 (0.35)	−0.37 (0.31)
Daily Friend	0.05(0.16)	−0.02 (0.19)	0.12 (0.24)	0.05 (0.49)	−2.43 (0.76) **	0.82 (0.65)	−0.15 (0.24)	−0.02 (0.18)	0.07 (0.24)
Homophily				−0.25 (0.55)	−0.96 (1.11)	−0.25 (1.23)			
Homophily × Daily Friend				−0.05 (0.60)	3.13 (1.01) **	−0.88 (0.76)			
Avg. Parent							1.34 (0.23) ***	1.07 (0.30) ***	1.71 (0.35) ***
Daily Parent							0.80 (0.15) ***	0.91 (0.20) ***	0.76 (0.21) ***
Avg. Parent × Daily Friend							−0.11 (0.16)	0.02 (0.18)	−0.22 (0.30)
Daily Parent × Daily Friend							−0.21 (0.31)	0.71 (0.44)	−0.63 (0.44)

’ *p* < 0.10, * *p* < 0.05, ** *p* < 0.01, *** *p* < 0.001. Note: Models control for youth gender. The total sample analyses also control for race–ethnicity; Avg. = average levels; Friend = Positive interactions with friends; Parent = Positive interactions with parents; Homophily = % of same-race–ethnicity friends.

**Table 5:** Mixed linear effects models examining associations between positive friend interactions and negative mood.

	Positive Friend Interactions			Positive Friend Interactions × Homophily			Positive Friend Interactions × Positive Parent Interactions		
	Total	Black	Latiné	Total	Black	Latiné	Total	Black	Latiné
	*B* (*SE*)	*B* (*SE*)	*B* (*SE*)	*B* (*SE*)	*B* (*SE*)	*B* (*SE*)	*B* (*SE*)	*B* (*SE*)	*B* (*SE*)
Intercept	2.20 (0.31) ***	2.52 (0.36) ***	1.64 (0.35) ***	2.66 (0.52) ***	3.15 (0.98) ***	2.15 (0.58) **	2.25 (0.30) ***	2.56 (0.36) ***	1.54 (0.30) ***
Avg. Friend	0.14 (0.20)	0.46 (0.30)	−0.16 (0.23)	0.02 (0.19)	0.22 (0.31)	−0.14 (0.23)	0.31 (0.21)	0.59 (0.34) ’	0.11 (0.21)
Daily Friend	−0.04 (0.12)	−0.06 (0.15)	−0.03 (0.17)	−0.04 (0.37)	−0.66 (0.67)	0.19 (0.47)	−0.07 (0.12)	−0.02 (0.16)	−0.02 (0.18)
Homophily				−0.94 (0.54) ’	−1.16 (0.89)	−0.71 (0.65)			
Homophily × Daily Friend				−0.12 (0.45)	0.46 (0.89)	−0.27 (0.55)			
Avg. Parent							−0.42 (0.20) *	−0.32 (0.28)	−0.73 (0.24) **
Daily Parent							−0.29 (0.12) *	−0.09 (0.18)	−0.39 (0.15) *
Avg. Parent × Daily Friend							−0.19 (0.13)	−0.41 (0.15) **	0.14 (0.22)
Daily Parent × Daily Friend							0.26 (0.25)	0.45 (0.39)	0.10 (0.32)

’ *p* < 0.10, * *p* < 0.05, ** *p* < 0.01, *** *p* < 0.001. Note: Models control for youth gender. The total sample analyses also control for race–ethnicity; Avg. = average levels; Friend = Positive interactions with friends; Parent = Positive interactions with parents; Homophily = % of same-race–ethnicity friends.

## Data Availability

The data presented in this study may not be available upon request from the DPW or MB due to privacy restrictions expected by the participants when they agreed to participate in this study.

## Note

1 The Little’s test conducted on the daily survey data rejected the null hypothesis that data is MCAR (X^2^ = 39.06, df = 11, *p* < 0.001). Consistent with the nature of daily diary methods, missingness is likely not unbiasedly distributed among participants and study days. We address possible biases with the use of full maximum likelihood estimation, and including random intercepts. To further assess for potential bias, we controlled for day of the study in our main effect model conducted on the total sample (i.e., Aim 1), and found that the inclusion of this covariate did not change our conclusions. Thus, we excluded it in the models presented in the manuscript.
